# Chemical Characterization and Ameliorating Effect of *Centratherum anthelminticum* Extract against Polycystic Ovary Syndrome in Wistar Rats

**DOI:** 10.1155/2023/4978562

**Published:** 2023-07-13

**Authors:** Moonis Shoaib, Ammara Saleem, Alam Zeb, Muhammad Imran Khan, Muhammad Furqan Akhtar

**Affiliations:** ^1^Riphah Institute of Pharmaceutical Sciences, Riphah International University Lahore, Lahore, Pakistan; ^2^Department of Pharmacology, Faculty of Pharmaceutical Sciences, GC University Faisalabad, Faisalabad, Pakistan; ^3^Department of Biochemistry, University of Malakand, Lower Dir, Khyber Pakhtunkhwa 18800, Pakistan

## Abstract

Polycystic ovary syndrome (PCOS) in females is an endocrine pathological condition of reproductive age which is usually caused by insulin resistance, hyperlipidemia, and oxidative stress. This research was aimed at evaluating the therapeutic effect of the *Centratherum anthelminticum* seed extract (CA) against PCOS in rodents as it is traditionally used to treat diabetes, inflammation, and gynecological problems. The CA was chemically characterized by high-performance liquid chromatography-diode array detection (HPLC-DAD). For the induction of PCOS, a high-fat diet (HFD) was given to all female Wistar rats for nine weeks except the normal control group, which was given a normal chow diet. Estradiol valerate was given to all rats except normal control. After the induction of PCOS, oral metformin (300 mg/kg) was given to the standard group, while CA was orally administered to diseased rats at 250, 500, and 750 mg/kg/day for 28 days. HPLC-DAD analysis revealed that kaempferol-3-pcoumaroylglucoside was present in the highest amount (146.8 ± 1.8 mg/g) of the extract followed by ferulic acid and malvidin-3-(6-caffeoyl)-glucoside. The *in vivo* results revealed a marked reduction in cholesterol and triglyceride levels in CA treatment groups. A significant rise was observed in progesterone and follicle stimulating hormone with a decrease in luteinizing hormone in the treatment groups as compared to disease control, which indicated normalization of the estrus cycle. The decrease in insulin resistance was characterized by low serum insulin levels in treatment groups. Treatment with CA also reduced inflammatory markers, such as IL-6 and NF-*κ*B in PCOS rats. NrF2 and oxidative stress markers such as catalase, superoxide dismutase, malondialdehyde, and reduced glutathione were also improved by CA in the ovary of diseased rats. Histopathological examination showed the different developmental stages of normal follicles in CA-treated diseased rats which were indicative of a normal fertile estrous cycle. Overall, the results confirmed the efficacy of CA against PCOS in treating estradiol-HFD-induced PCOS due to its antidiabetic, anti-inflammatory, antihyperlipidemic, and antioxidant properties.

## 1. Introduction

Polycystic ovary syndrome (PCOS), a reproductive age endocrine disorder, affects 5.6–21.3% of the female population among all ethnicities [1–3]. PCOS is a heterogenous disorder, with a range of different phenotypes, which results in differences in clinical opinions and difficulties in the diagnosis and treatment [4]. The key features of PCOS include hyperandrogenism, ovulatory dysfunction, acne, obesity, menstrual disturbance, mood swings, fertility challenges, hirsutism, androgenetic alopecia, and acanthosis nigricans. Hormonal, environmental and genetic factors influence PCOS etiology [5]. Women with PCOS are at a higher risk of cardiometabolic comorbidities, which encompass cardiovascular disorders, nonalcoholic fatty liver disease, lipid disorders, and diabetes mellitus in addition to depression, anxiety, weight gain, and bulimic behavior [6]. Pharmacological interventions include oral antidiabetics and oral contraceptives, which have the potential of developing not only obesity but also metabolic abnormalities [7, 8]. Estrogen receptor modulators, i.e., clomiphene citrate and aromatase inhibitors, i.e., anastrozole, or recombinant follicle stimulating hormone (FSH) injections are given for the induction of ovulation in PCOS patients which lead to numerous pregnancies [9].

Abnormally elevated levels of androstenedione, insulin, luteinizing hormone (LH), and serum testosterone are evident in PCOS patients [10, 11]. Hyperandrogenism plays a pivotal role in 90% of PCOS individuals [12]. Excessive androgen production is caused by hypothalamic-pituitary-ovarian axis dysfunction, oxidative stress, insulin resistance, and steroidogenesis abnormalities, which leads to PCOS-related symptoms [9, 13]. Gonadotropin-releasing hormone originating from hypothalamus causes release of FSH and LH from anterior pituitary gland. FSH is involved in initiation and development of gametogenesis, whereas LH causes maturation of follicles and ovulation, in part by induction of various hormones involved in these developments [14, 15].

Multiple studies in rodent models of PCOS demonstrated that several herbs might be beneficial for the treatment of laboratory and clinical symptoms of PCOS, such as insulin resistance, menstrual disturbances, infertility, hormonal status, and anthropometric indices. These herbs and their extracts can be either used alone or in combination with standard treatments [8]. *Centratherum anthelminticum* (CA) Kuntze (bitter cumin/black cumin) is a member of the Asteraceae family and is commonly used in Ayurvedic medicine. Over 120 chemical compounds have been discovered in different plant parts of CA. It is rich in phytochemicals, particularly butin, tetrahydroxy chalcone, and tetrahydroxy flavone [16]. Ayurvedic medicine reveals that CA helps in healing and alleviating diabetes. Moreover, this plant is popular for being an astringent, anthelmintic, tonic, treating fever, skin problems, kidney stones, and diuretic activity. Recently, the anticancer property of the active compound from CA seeds has also been established [17].

Previous studies demonstrated that the CA had exhibited a reduction in reactive oxygen species (ROS) and lipid peroxidation and improved the antioxidant enzyme activities, including superoxide dismutase (SOD) and catalase (CAT) to protect the damage to macromolecules such as DNA, membrane lipids, and proteins. Moreover, CA caused a significant decrease in both the cardiac and liver function biomarkers associated with intracellular microvesicular steatosis and lipid accumulation [18]. Multiple studies on CA showed its activity against diabetes and related complications due to antihyperlipidemic and antioxidant properties. As PCOS is associated with ROS, hyperlipidemia, and insulin resistance, therefore, CA could exhibit anti-PCOS activity in Wistar rats. Hence, the aim of this study was to explore the curative effect of CA against PCOS in Wistar rats.

## 2. Materials and Methods

### 2.1. Chemicals and Equipment

Estradiol valerate (Bayer-Schering Pharma, Pakistan), hydrogen peroxide (H_2_O_2_), sodium chloride (NaCl), chloroform (CHCl_3_), sodium hydroxide (NaOH), sodium phosphate buffer solution (PBS), pyrogallol, potassium phosphate (K_3_PO_4_), trichloroacetic acid (TCA), thiobarbituric acid (TBA), hydrochloric acid (HCl), monosodium phosphate, disodium phosphate, dithiobis nitrobenzoic acid (DTNB), and sodium potassium tartrate were obtained from Sigma-Aldrich, USA. HPLC-DAD (Agilent 1260 Infinity, Germany), double beam spectrophotometer (Shimadzu UV-1601 Japan), Glucometer AccuChek® Performa (Roche Diabetes Care), and rotary evaporator (RE-501-Germany) were used in the study.

### 2.2. Ethanol Extract Preparation

The plant was collected from Punjab and identified by a taxonomist at the University of Punjab (Ref. No. LAH#61120B). Dried seeds of *C. anthelminticum* were ground, and the ethanolic extract was made by soaking the coarsely powdered seed in ethanol at 1 : 10. The soaked material was kept for 14 days at room temperature in an amber-colored flask and then passed through Whatman filter paper No. 43 for filtration. The filtrate was concentrated in a rotary vacuum evaporator at 40°C. The brown-colored*C. anthelminticum* ethanolic seed extract (CA) was kept in the refrigerator at 2–8°C till further use.

### 2.3. HPLC-DAD Analysis

A solution of 100 mg extract/10 ml of methanol was prepared and filtered through a syringe filter into the HPLC vials (2 mL). The injection volume was 50 *μ*L of the sample solution was injected and analyzed by HPLC-DAD, according to the previous method [19]. The C18 column was used for the analysis.

### 2.4. Experimental Animals

Thirty-six nulliparous female Wistar rats of weight ranging from 120 to 150 g were obtained and kept at the animal house of Riphah International University. All rats were kept at 25 ± 3°C and 55–65% humidity during acclimatization and experimental study. All protocols were executed as per the guidelines of the Institutional Animal Ethical Committee (Ref. No. REC/RIPS-LHR/2022/058).

### 2.5. Induction of PCOS

High-fat diet (HFD) comprising 40% fat, 20% protein, 36% carbohydrate, and 4% others (530 kcal) was given to all animals for nine weeks, except normal control (NCG) which received normal rodent chow diet [20]. Estradiol valerate (4 mg/kg) was given orally once at the start of feeding the female rats with HFD for the induction of PCOS. The animals with a 10–20% increase in weight than the normal control group were considered obese [21, 22]. To confirm the development of PCOS, vaginal smears were taken to observe estrous cycle. Moreover, three rats were randomly selected and evaluated at the 9^th^ week by removing their ovaries for histological examination and compared with that of NCG [21].

### 2.6. Study Design

Rats on a normal chow diet served as NCG. All animals except NCG received HFD and estradiol valerate and were divided into five groups. Disease control (DCG) received distilled water, while animals in the standard therapy group (STG) orally received metformin 300 mg/kg/day [23]. The remaining three animal groups were orally treated with CA 250, 500, or 750 mg/kg/day. Treatment with different therapies was started after 9 weeks of starting HFD and then continued for 28 days [24].

### 2.7. OGTT, Lipid, and Liver Function Parameters

The blood was taken from the rat tail for an oral glucose tolerance test (OGTT) on the 28^th^ day. For performing OGTT, 75 mg/ml glucose solution was administered via oral route to each animal and blood glucose level was checked at 0, 30, 60, 90, 120, and 180 min after glucose administration. Triglycerides and total cholesterol were estimated by using ELISA kits. Moreover, the blood sample was pulled out after 28 days of therapy by cardiac puncture under anesthesia with chloroform. Blood was collected in ethylenediaminetetraacetic acid (EDTA) tubes. Serum was collected by centrifugation of blood at 1000 rpm and stored in a refrigerator for further use. Liver function tests i.e., glutamic-pyruvic transaminase (SGPT) and glutamic oxaloacetic transaminase (SGOT) were analyzed by standard methods [25].

### 2.8. Histological Examination

For histopathological studies, the cervical dislocation technique was used to kill the anesthetized animals and ovaries were dissected out through abdominal excision and placed in 10% buffered formaldehyde. The ovarian tissues were fixed in paraffin wax and sections of 4-5 *μ*m were prepared with a microtome. The excised ovarian tissues were stained with hematoxylin and eosin for histological examination under a light microscope [26].

### 2.9. Measurement of Sex Hormones and Inflammatory Markers

The ELISA kits of FSH, LH, progesterone, nuclear factor erythroid 2-related factor 2 (NrF2), interleukin (IL-6), and nuclear factor kappa B (NF-*κ*B) by PerkinElmer Health Sciences, Inc., USA, were utilized in the experimental study for rats [21].

### 2.10. Preparation of Tissue Homogenates

For tissue homogenate preparation, ovaries were thoroughly rinsed with ice-cold phosphate buffer saline (PBS). The ovarian tissues were thoroughly crushed with a homogenizer. The PBS was added to the crushed tissues to make 10% w/v homogenate. Afterward, the mixtures were centrifuged for 5 min at 1000 rpm, and supernatants were collected to determine oxidative stress parameters [27].

### 2.11. CAT Activity

CAT activity was estimated by a previously established method. A cuvette was filled with 1 ml supernatant which already contained 1.95 ml phosphate buffer (50 mM, pH 7.0) and 1 ml 30 mM H_2_O_2_. Absorbance change was measured for 30 S at a wavelength of 240 nm. The CAT activity was stated in U/mg of protein. Indeed, one CAT activity unit was equal to 1 M of hydrogen peroxide decomposed per min at 25°C [28].

### 2.12. MDA Level

The MDA is a lipid peroxidation index that is determined by the double heating method. This method is based upon the measurement of absorbance of purple color produced by the TBA reaction. To carry out this estimation, 2.5 ml TCA was mixed with 0.5 ml supernatant. Then, these solutions were kept in a boiling water bath for 15 min followed by cooling down to room temperature. Centrifugation was carried out at 1000 rpm for at least 10 min. Collected supernatant (2 ml) was admixed with 1 ml TBA solution (0.67%, w/v). These solutions were again boiled over a water bath for 15 min. The absorbance was calculated at 532 nm after cooling [29].

### 2.13. SOD Activity

Free radical scavenging capacity in the tissue homogenates was measured. These radicals were produced by pyrogallol auto-oxidation. The reaction mixture was comprised of 2.8 ml PBS, 100 *µ*l pyrogallol solution (2.6 mM in 10 mM HCl), and 100 *µ*l sample supernatant. Absorbance was recorded repeatedly at 325 nm after every 30 S. Under the assay conditions, each unit of SOD was equal to the quantity of enzyme needed to achieve 50% pyrogallol autoxidation inhibition [30].

### 2.14. GSH Level

For GSH estimation, a tissue homogenate supernatant of 1 ml was mixed up with an equal volume of 10% TCA. To this mixture, 1 ml phosphate solution was added along with 0.5 ml DTNB reagent was added. Absorbance was taken at 412 nm. The values were stated as nm of GSH per mg of protein [31].

### 2.15. Statistical Analysis

A one-way analysis of variance (ANOVA) test was applied to the data regarding hormonal, lipid and liver parameters, insulin, HbA1c, oxidative stress, and inflammatory parameters using GraphPad Prism 5 (CA, USA, San Diego). Changes in body weight were assessed by two-way ANOVA. Moreover, the statistically significant data were subjected to Tukey's post hoc test. The results were moderately significant when *p* < 0.01, while these were considered highly significant when *p* < 0.001.

## 3. Results

### 3.1. Chemical Composition

**Figure 1:**
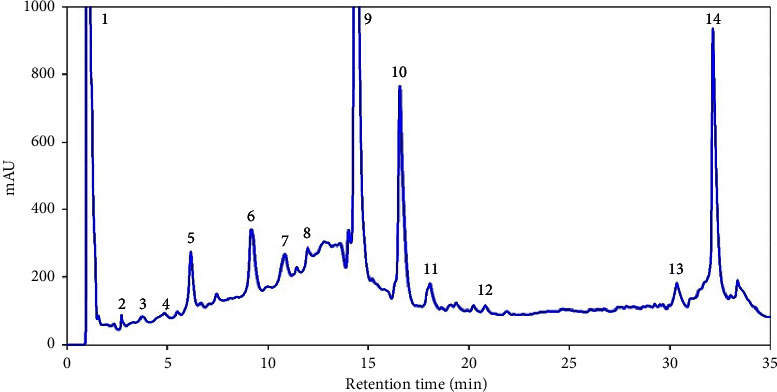
HPLC-DAD spectra of *Centratherum anthelminticum* seed extract.

HPLC-DAD analysis revealed that kaempferol-3-pcoumaroylglucoside was present in the highest amount (146.8 ± 1.8 mg/g) of the extract followed by ferulic acid (145.8 ± 1.21 mg/g) and malvidin-3-(6-caffeoyl)-glucoside (77.1 ± 0.4 mg/g). The detailed phytochemical analysis of the CA extract is shown in Table 1, while the HPLC-DAD spectrum is presented in Figure 1.

### 3.2. Effect on Blood Glucose, Insulin, and Body Weight

#### 3.2.1. OGTT

**Figure 2:**
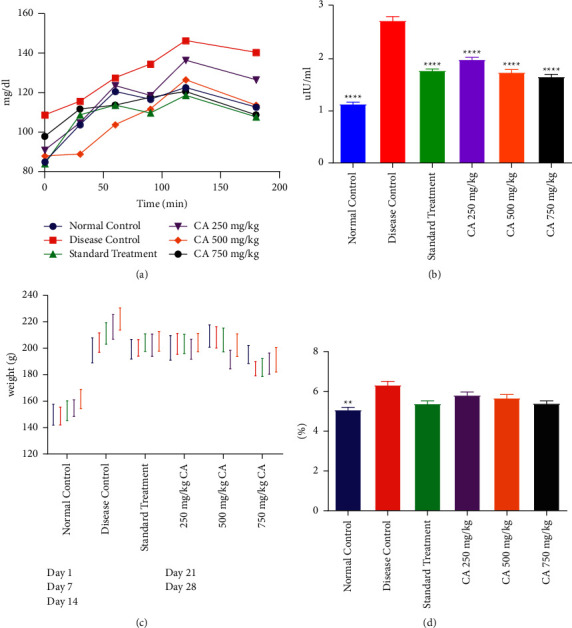
Effect of *Centratherum anthelminticum* extract (CA) on serum glycemic markers and body weight in PCOS rats. Effect of CA on (a) OGTT, (b) insulin, (c) body weight, and (d) glycated hemoglobin. Data displayed as mean ± S.D. (*n* = 6). ^*∗∗∗∗*^*p* < 0.0001 and ^*∗∗*^*p* < 0.01 showed statistical difference when compared with the disease control group.

Blood glucose level via OGTT was recorded in all groups at the end of the treatment which revealed that the STG and all different doses of CA decreased the blood glucose levels 90 min after glucose administration. The DCG demonstrated the highest rise in blood glucose. The effect of CA 250 mg/kg on blood glucose levels was minimum among all therapeutic groups. The effect of CA on blood glucose levels on OGTT in PCOS rats is shown in Figure 2(a).

#### 3.2.2. Serum Insulin Levels

Administration of estradiol and HFD led to a significant increase in insulin level (hyperinsulinemia) of PCOS animals as compared to NCG. The PCOS rats treated with STG and different doses of CA demonstrated a significant decrease (*p* < 0.0001) in insulin as compared to DCG. The effect of treatment with different doses of CA on the serum insulin of PCOS rats is shown in Figure 2(b).

#### 3.2.3. Effect on Body Weight

All animals had statistically indifferent body weights at the time of initiation of the experiment. With HFD for 9 weeks, there was a significant rise in body weight of almost 10–20 percent among all animals as compared to NCG. There was a continuous rise in the body weight of DCG. All treated groups demonstrated higher body weight as compared to the NCG. The effect of treatment with different doses of CA on the body weight of PCOS rats is shown in Figure 2(c).

#### 3.2.4. HbA1c Level

It was found that the DCG had a significantly increased level of HbA1c as compared to NCG. No significant difference in HbA1c was observed in the diseased animals treated with metformin or CA compared with DCG. The effect of CA groups was comparable with the DCG. The effect of treatment with different doses of CA on the HbA1c of PCOS rats is shown in Figure 2(d).

### 3.3. Effect on Serum Lipids and Liver Enzymes

#### 3.3.1. Serum Triglycerides

**Figure 3:**
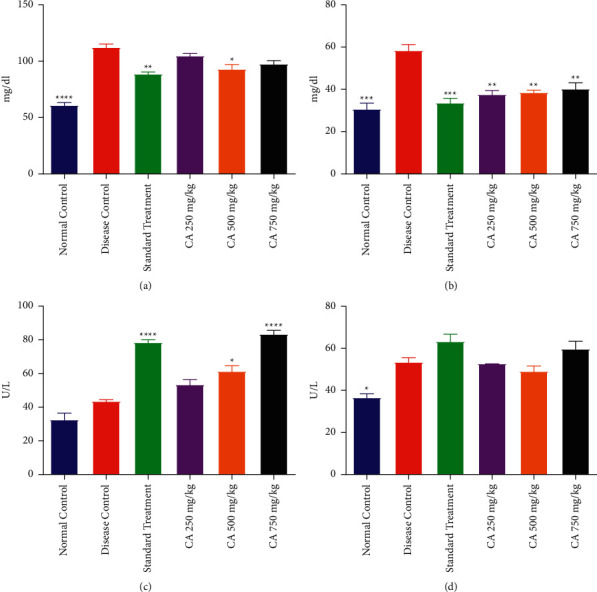
Effect of *Centratherum anthelminticum* extract (CA) on lipid profile and liver function in PCOS rats. Data displayed as mean ± S.D. (*n* = 6). ^*∗∗∗∗*^*p* < 0.0001 and ^*∗∗∗*^*p* < 0.001 and ^*∗∗*^*p* < 0.01 and *p* < 0.05 showed statistical difference when compared with the disease control group. (a) Triglycerides, (b) cholesterol, (c) SGOT, and (d) SGPT.

Triglyceride level was increased in the DCG as compared to NCG after administration of estradiol and HFD. The treatment groups receiving metformin or CA 500 mg/kg demonstrated a decrease in triglycerides as compared to DCG. The effect of treatment with different doses of CA on the triglyceride level of PCOS rats is shown in Figure 3(a).

#### 3.3.2. Serum Cholesterol

Hyperlipidemia is one of the main causes of PCOS. Serum cholesterol level was observed in all groups which showed that DCG had elevated levels of cholesterol as compared to NCG. The STG and CA at all dose levels significantly reduced the cholesterol level as compared to DCG. The effect of treatment with different doses of CA on the serum cholesterol of PCOS rats is shown in Figure 3(b).

#### 3.3.3. SGOT Level

The level of SGOT was insignificantly different among DCG as compared to NCG. However, treatment with metformin and CA 500 and 750 mg/kg significantly raised the level of SGOT as compared to DCG. The treatment group CA 250 mg/kg when compared with the STG displayed a statistically comparable level of SGOT as compared to NCG and DCG. The effect of treatment with different doses of CA on the SGOT level of PCOS rats is shown in Figure 3(c).

#### 3.3.4. SGPT Level

It was found that the DCG showed a statistically elevated level of SGPT as compared to NCG. However, treatment with metformin and CA did not result in any significant changes in SGPT as compared to DCG. The effect of treatment with different doses of CA on the SGPT level of PCOS rats is shown in Figure 3(d).

### 3.4. Effect on Reproductive Hormones

#### 3.4.1. FSH Level

**Figure 4:**
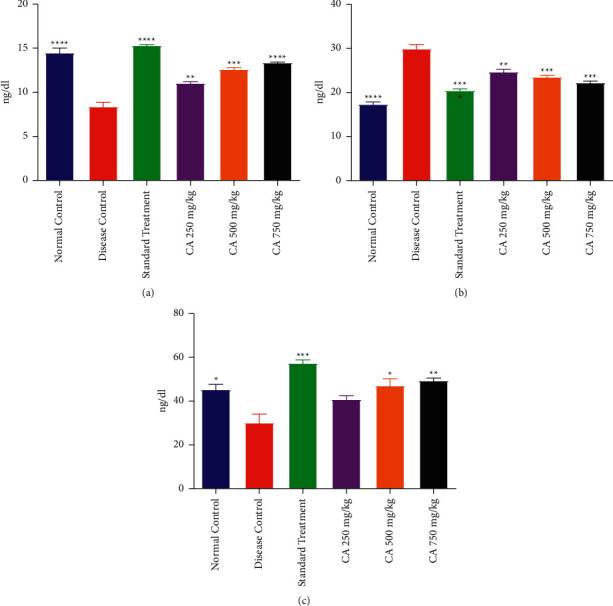
Effect of *Centratherum anthelminticum* extract on hormones in PCOS rats. Data displayed as mean ± S.D. (*n* = 6). ^*∗∗∗∗*^*p* < 0.0001 and ^*∗∗∗*^*p* < 0.001 and ^*∗∗*^*p* < 0.01 and *p* < 0.05 showed statistical difference when compared with the disease control group. (a) FSH, (b) LH, and (c) progesterone.

The level of FSH declined in the DCG as compared to NCG. However, the treatment of PCOS rats with CA or metformin significantly increased the level of FSH in comparison with DCG. The treatment with CA 750 mg/kg exhibited the maximum increase in FSH; however, the effect on FSH was lower than that of metformin. The effect of treatment with different doses of CA on the FSH level of PCOS rats is shown in Figure 4(a).

#### 3.4.2. LH Level

The LH level was increased in the PCOS rats of DCG as compared to NCG. The STG and CA-treated groups demonstrated a significantly reduced level of LH as compared to DCG. However, the level of LH in STG and DCG was still elevated as compared to NCG. The effect of treatment with different doses of CA on the LH level of PCOS rats is shown in Figure 4(b).

#### 3.4.3. Progesterone Level

The progesterone level declined in the PCOS rats of DCG as compared to NCG. Administration of CA at 500 and 750 mg/kg and metformin resulted in a significant elevation of progesterone as compared to DCG. The effect of CA 250 mg/kg was minimal, and the level of progesterone was insignificant to DCG. The effect of treatment with different doses of CA on progesterone level of PCOS rats is shown in Figure 4(c).

### 3.5. Effect on Inflammatory Markers

#### 3.5.1. IL-6 Level

**Figure 5:**
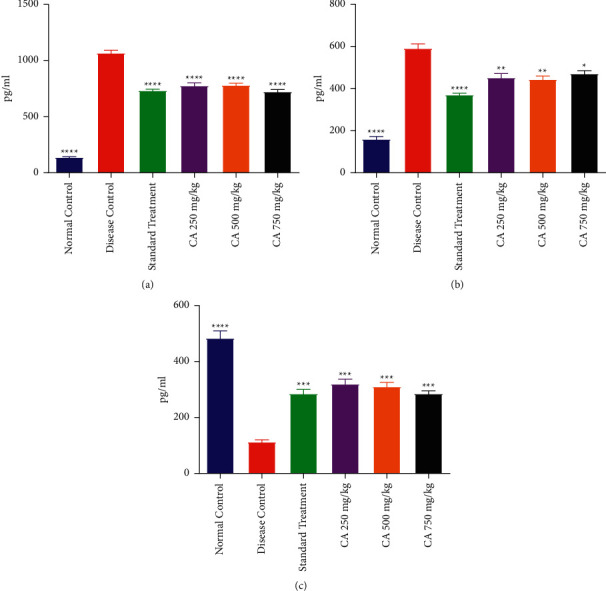
Effect of *Centratherum anthelminticum* extract (CA) on IL-6, NF-*κ*B, and NrF2 in PCOS rats. (a) IL-6, (b) NF-*κ*B, and (c) NrF2. Data displayed as mean ± S.D. (*n* = 6). ^*∗∗∗∗*^*p* < 0.0001 and ^*∗∗∗*^*p* < 0.001 and ^*∗∗*^*p* < 0.01 and *p* < 0.05 showed statistical difference when compared with the disease control group.

It was found that the administration of estradiol and HFD resulted in a significant elevation of IL-6 in DCG as compared to NCG. Treatment with CA at all dose levels and STG significantly declined IL-6 level as compared to DCG. However, CA and STG treated rats demonstrated a higher level of IL-6 in comparison to NCG. The effect of treatment with different doses of CA on the IL-6 level of PCOS rats is shown in Figure 5(a).

#### 3.5.2. NF-*κ*B Level

This study showed that DCG exhibited a higher level of NF-*κ*B level in the serum in comparison to the NCG. Treatment of PCOS rats with CA at 350, 500, and 750 mg/kg resulted in a statistically significant decline in NF-*κ*B as compared to DCG. However, the treatment of PCOS rats with CA and metformin did not result in the normalization of NF-*κ*B. The effect of treatment with different doses of CA on NF-*κ*B levels in the serum of PCOS rats is shown in Figure 5(b).

#### 3.5.3. NrF2

It was demonstrated that the administration of estradiol and HFD had resulted in a significant decline of NrF2 in DCG as compared to NCG. Treatment with CA at all dose levels and STG significantly raised the NrF2 level as compared to DCG. The effect of treatment with different doses of CA on NrF2 levels in the serum of PCOS rats is shown in Figure 5(c).

### 3.6. Oxidative Stress in the Ovary

#### 3.6.1. CAT Activity

**Figure 6:**
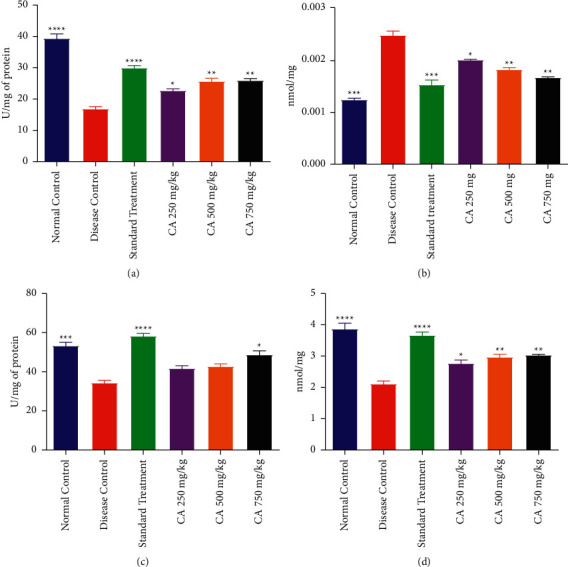
Effect of *Centratherum anthelminticum* extract (CA) on oxidative stress in ovary of PCOS rats (a) catalase, (b) malondialdehyde, (c) superoxide dismutase, and (d) reduced glutathione. Data displayed as mean ± S.D. (*n* = 6). ^*∗∗∗∗*^*p* < 0.0001 and ^*∗∗∗*^*p* < 0.001 and ^*∗∗*^*p* < 0.01 and *p* < 0.05 showed statistical difference when compared with the disease control group.

It was observed that the activity of CAT in the ovary tissue homogenate of PCOS rats had reduced significantly as compared to NCG. In the STG, there was a significant increase in CAT activity (*p* < 0.0001) in ovaries as compared to the DCG. Although, all PCOS animals treated with CA showed a significant increase in CAT activity as compared to DCG; however, the CA 500 and 750 mg/kg exhibited a higher level of CAT as compared to the lowest dose of CA. The effect of treatment with different doses of CA on CAT activity in the ovarian tissue of PCOS rats is shown in Figure 6(a).

#### 3.6.2. Malondialdehyde Level

The MDA level in DCG was significantly higher than the NCG. The treatment groups receiving CA at 250, 500, and 750 mg/kg or STG displayed a significant decrease in the MDA level as compared to DCG. The effect of CA at 500 and 750 mg/kg or STG on MDA was comparable to that of NCG. The effect of treatment with different doses of CA on MDA levels in the ovarian tissue of PCOS rats is shown in Figure 6(b).

#### 3.6.3. SOD Activity

A significant decline of SOD activity in the ovarian tissue of DCG was observed as compared to NCG. Administration of metformin or CA 750 mg/kg significantly elevated the activity of SOD in diseased rats as compared to DCG. However, CA 250 and 500 mg/kg treatments failed to bring out any significant increase in the SOD activity of diseased rats in comparison with the DCG. The effect of treatment with different doses of CA on SOD activity in the ovarian tissue of PCOS rats is shown in Figure 6(c).

#### 3.6.4. GSH Level

The level of GSH was lower in the estradiol-induced PCOS rats in comparison with the NCG. The STG and CA 250, 500, and 750 mg/kg treatments in diseased rats significantly increased the level of GSH as compared to DCG. However, the level of GSH exhibited by CA 250 and 500 mg/kg groups was significantly lower than that displayed by NCG. The effect of treatment with different doses of CA on GSH levels in the ovarian tissue of PCOS rats is shown in Figure 6(d).

#### 3.6.5. Histopathological Examination of Ovaries

**Figure 7:**
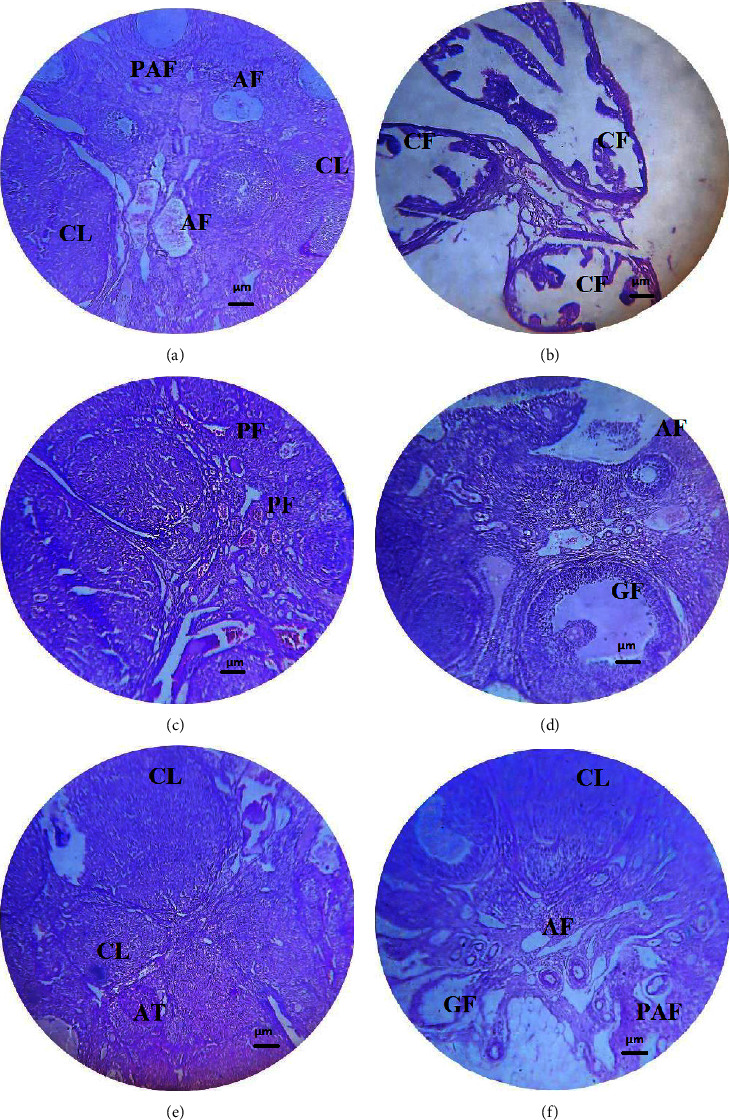
Histopathology of PCOS rat ovary treated with *Centratherum anthelminticum* extract at 40x magnification. (a) Section of the ovary of the normal control group showing follicles, (b) ovarian section from the disease control group showing multiple cystic follicles, (c) ovarian section treated with metformin showing primary follicles, (d) ovarian section treated with 250 mg/kg plant extract showing large follicle, (e) ovarian section treated with 500 mg/kg showing different corpus luteum stages, and (f) ovarian section treated with 750 mg/kg showing different development follicles stages. AF: antral follicle; PAF: preantral follicle; AT: atretic follicle; PF: primary follicle, CF: cystic follicle; CL: corpus luteum; and GF: graafian follicle.

Histopathological slides of the ovary of all PCOS rats treated with metformin and CA extract were examined and compared with DCG and NCG for the number and size of follicles and the effect on the corpus luteum. In the NCG, the maximum number of corpus luteum and primary follicles i.e., preantral follicles (PAF) and antral follicles (AF) were observed. Changes in numbers of AF were also observed in the CA 250 mg/kg treated group, along with shrinkage of ovary tissues and decreased number of corpus luteum. Primary and secondary follicles were also seen. However, the most pronounced changes were observed in PCOS rats treated with CA 750 mg/kg group which demonstrated several follicles such as graafian follicle (GF), AF, and PAF at different developmental stages. Corpus luteum was also observed with decreased number of secondary follicles, thus exhibiting the restoration of fertility as shown in Figure 7.

## 4. Discussion

Like other endocrine diseases, PCOS is treated by lifestyle modifications and pharmacological and hormonal therapy. Metformin has established its role in the management of PCOS over time, but due to its deleterious effects, pharmacological interventions with multiple herbal extracts have been assessed which showed efficacy alone or in combination with conventional therapy against PCOS [8]. In current study, the plant extract was chemically characterized with HPLC-DAD to determine the phytochemical composition. The effect of CA at 250, 500, and 750 mg/kg and the standard metformin therapy on PCOS rats was observed which demonstrated that these treatments caused amelioration of PCOS symptoms through effects on lipid profile, insulin levels, liver function, and serum hormones. The histopathological observations validated the preclinical evidence.

Different phytochemicals including kaempferol, ferulic acid, malvidin, caffeoylquinic acid, and quercetin derivatives in the plant extract were detected. These components extracted from the plants have different pharmacological effects, including anti-inflammatory, antipyretic, anthelmintic, antioxidant, antimicrobial, antiviral, anticancer, antifilarial, wound healing, and diuretic activity [32, 33].

Insulin plays a vital role in stimulating stromal androgen and ovarian thecal secretion. Insulin resistance, in the form of hyperinsulinemia, induces the overproduction of ovarian androgens. Moreover, terminal differentiation and anovulation are induced by the impact of insulin-like growth factor (IGF)-1 on LH. The IGF-1 enhances the production of estrogen through granulosa cells and acts in regulating aromatase concentration of granulosa cells via synergism with LH and FSH [34, 35]. Insulin resistance, an important factor for the diagnostic criteria of PCOS, is usually treated with metformin. Treatment with CA improved insulin resistance as evidenced by a reduction of insulin level in PCOS rats [8]. The phytochemicals in CA, such as quercetin glycoside, kaempferol, and ferulic acid, have antioxidant, anti-inflammatory, and antidiabetic effects. Furthermore, there are multiple possible mechanisms behind the antidiabetic action of CA, such as inhibition of carbohydrate hydrolyzing enzyme activity, including *α*-amylase and *α*-glucosidase, which decrease glucose absorption in the gastrointestinal tract [17].

Steroidal hormones play a pivotal role in sexual maturation and differentiation and are synthesized from cholesterol under the influence of gonadotrophins. These steroidal hormones are produced in steroidogenic tissues involving six different cytochrome P450 enzymes [36]. These gonadotrophins are scanty in PCOS which result in reduced steroidogenesis and failure of egg production/maturation [37, 38]. In PCOS women, the rate of adrenal and ovarian androgen production is significantly increased, while their metabolic inactivation is reduced. In addition, adipose tissues secrete adipocytokines such as leptin and chemerin, which aggravate PCOS. The decrease in body weight reduces the secretion of adipocytokines and insulin resistance, while correction of the gonadotropin hormone release from the pituitary gland corrects the steroidal hormone homoeostasis [38].

The present study showed that the CA had resulted in a reduction of cholesterol and triglyceride level in PCOS rats. The effect of CA on cholesterol might be either due to the inhibition of the *ß*-hydroxy *ß*-methylglutaryl CoA reductase (HMG-CoA), the enzyme important for rate-limiting step in the biosynthesis of cholesterol, or an increase in the low-density lipoprotein (LDL-c) breakdown through its receptors on the liver. The decrease in triglycerides may be related to the augmented activity of the endothelial-bound lipoprotein lipase, which leads to the hydrolysis of triglycerides into fatty acids [18]. Hyperlipidemia is directly linked to oxidative stress and insulin resistance. The antihyperlipidemic and antioxidant properties of *C. anthelminticum* have been established in multiple studies. Previously, it is demonstrated that *C. anthelminticum* had improved the oxidative stress and lipid profile in triton X-100-induced hyperlipidemic rabbits [18].

The administration of HFD and estradiol influenced the hormonal levels. The effect of estradiol and HFD was evident on the liver as diseased rats showed a significant rise in levels of SGPT and SGOT. This study has shown the ability of CA to regulate the hormonal imbalance caused by the endocrine disorder which had caused an increase in LH and a decrease in progesterone and FSH. It demonstrated the ability of CA to prevent the progression of PCOS.

Oxidative stress induced due to hyperlipidemia is one of the associating factors that point to long-term complications of PCOS by enhancing free radical production and suppressing the antioxidant enzyme system in the body [36]. The oxidative modification of LDL-c due to stress plays a major part in atherosclerotic lesions [18]. This study showed that CA reduced oxidative stress to prevent long-term complications of PCOS, i.e., atherosclerosis.

It is found that the reduction of SOD and CAT activities and increase in MDA level are the results of oxidative imbalance in reproductive tissues. Oxidative stress in ovarian tissues of PCOS rats is evident in previous studies. Long-term oxidative stress leads to sperm immobility and damage to DNA, enzymes, and lipid peroxidation [37–39]. There was an elevation of the SOD activity by CA 750 mg/kg in PCOS rats. The decrease in SOD activity in untreated PCOS rats showed oxidative stress induced by HFD and estrogen. An important antioxidant having the ability to protect the cells against oxidative stress is GSH. Optimum levels in the GSH/GSSG ratio produce a reduced environment leading to protection from radical oxygen species [40]. In the present research, the GSH levels were restored in ovaries in CA 500 mg/kg/day and 750 mg/kg treatment groups. CAT is an inducible enzyme that converts hydrogen peroxide into water and oxygen. CAT activity was reduced in diseased animals, while treatment with CA significantly increased the CAT activity to protect ovarian tissue. The increase in MDA level is indicative of lipid peroxidation which is evident in PCOS induced by HFD and estradiol. Current study showed that CA reduced the lipid peroxidation in diseased rats at different dose levels.

Multiple shreds of evidence supported that high levels of proinflammatory markers TNF*α*, IL-6, NF-*κ*B, and IL-1*ß* are associated with PCOS [41]. An elevated level of IL-6 is not termed as a prominent feature of PCOS but can be considered as a beneficial surveillance biomarker for PCOS treatment [42]. PCOS women with low-grade chronic inflammation might be at risk of cardiovascular diseases and diabetes mellitus due to chronic inflammation [43]. This study demonstrated that the treatment with CA resulted in the decline of serum levels of NF-*κ*B, NrF2, and IL-6 in PCOS rats which resulted in a reduced risk of chronic inflammation.

Histopathological substantiation in the current study showed that the ovaries of HFD and estradiol valerate-treated rats exhibited numerous cysts along with either a very lean or no granulosa layer. Moreover, diseased animals showed anovulation which was indicated by the absence of the corpus luteum. Treatment with CA led to the cyst disappearance and emergence of corpora lutea and normal follicles. Sections from CA 250 mg/kg treatment showed follicles bigger in size and lesser corpora luteum. CA at 759 mg/kg showed several corpus luteum and antral follicles with clearly differentiate oocytes, granulosa cell layer, corona radiate, cumulus oophores, and thecal cells indicating improvement of PCOS [44].

## 5. Conclusion

Based on the current study, it can be concluded that the extract of *C. anthelminticum* displayed an ameliorating effect against PCOS in rats due to anti-inflammatory, antioxidant, antihyperlipidemic, and antidiabetic activities. The plant extract was the most efficacious at 750 mg/kg. These *in vivo* activities were most probably due to the presence of flavonoids and phenolic compounds which decreased the oxidative stress, insulin resistance, and HbA1c and normalized the hormonal imbalance to restore ovulation.

It is suggested that the plant extract must be evaluated for its antiobesogenic potential in diabetic rats. Furthermore, a comprehensive phytochemical analysis and bioassay-based isolation of the multiple phytochemicals responsible for these activities must be carried out to discover a safer, efficacious, and economical treatment of PCOS.

## Figures and Tables

**Table 1:** Phenolic profile of *Centratherum anthelminticum* ethanolic extract (mg/g).

Peak	Rt (min)	Identity	Mean	STD
1	1	Ferulic acid	145.8	1.21
2	2.7	Sinapoyl hexoside	4.1	0.05
3	3.7	3-Caffeoylquinic acid	8.0	0.19
4	4.8	5-Caffeoylquinic acid	13.0	0.15
5	6.1	4-Caffeoylquinic acid	21.2	0.2
6	9.1	3,5-Di-caffeoylquinic acid	36.5	0.2
7	10.8	4,5-Di-caffeoylquinic acid	29.7	0.1
8	11.9	3-Feruloyl-5-caffeoylquinic acid	22.7	0.1
9	14.3	Kaempferol-3-pcoumaroylglucoside	146.8	1.8
10	16.6	Di-caffeoylquinic acid	45.9	1.8
11	18.1	Quercetin-3-sinapolysophoroside-7-glucoside	34.9	0.0
12	20.8	Isorhamnetin-3-sophoroside-7-glucoside	10.1	0.1
13	30.3	Malvidin-3-(6-caffeoyl)-glucoside	77.1	0.4
14	32.1	Peonidin-3-(6″-*p*-coumaroyl)-glucoside	50.0	0.9
Total			665.5	

## Data Availability

The data used to support the findings of this study are available from the corresponding author upon request.
